# Application of key link nursing based on feedforward control theory in the emergency rescue of multiple trauma patients

**DOI:** 10.3389/fpubh.2025.1707141

**Published:** 2025-12-17

**Authors:** Yufeng Huang, Fei Yin, Yuehua Ni, Ying Zhang, Chen Dai

**Affiliations:** Department of Emergency, Ninth People’s Hospital of Suzhou City, Suzhou, China

**Keywords:** emergency multiple injury, feedforward control theory, key link nursing, rescue, satisfaction with care

## Abstract

**Objective:**

To explore the application effect of key link nursing, which is based on feedforward control theory, in the rescue of emergency patients with multiple injuries.

**Methods:**

A total of 120 patients with multiple injuries admitted to the emergency department of Suzhou Ninth People’s Hospital from August 2022 to February 2025 were included in the study and grouped according to different emergency modes: 46 patients with multiple injuries were included in the conventional group (conventional emergency care), and 74 patients were included in the feedforward control group (nursing intervention in key links on the basis of feedforward control theory). The emergency time, treatment effect, patient family nursing satisfaction, complication rate and trauma severity of the feedforward control group and the conventional group were compared.

**Results:**

Compared with those in the conventional group, the time to establish intravenous access, the time to complete systemic examination, the time to stay in the emergency department, the time from the emergency department to the operating room, the incidence rates of MODS and DIC, the referral rate and the incidence of complications in the feedforward control group were significantly lower. Compared with those of the conventional group, the process handling, communication explanation, rescue skills, prepreparation score, nursing satisfaction score and scores of each dimension of the SF-36 significantly increased (*p* < 0.05). The proportion of severe injuries in the feedforward control group after treatment was significantly lower than that in the conventional group (*p* < 0.05).

**Conclusion:**

Key link nursing based on feedforward control theory can improve the rescue success rate of emergency patients with multiple injuries, shorten the rescue time, improve the severity of patients’ injuries, reduce the occurrence of complications, effectively improve management satisfaction, and improve patients’ quality of life.

## 1 Introduction

Traumatic injuries represent a formidable global public health challenge, causing approximately 4.4 million deaths worldwide in 2019 and accounting for nearly 8% of all deaths (1). This burden is disproportionately borne by low- and middle-income countries, where mortality rates are substantially higher (2). The leading etiologies globally include road traffic injuries, falls, and interpersonal violence (3). In China, the scale of the issue is similarly significant, with injuries responsible for 7.51% of all-cause deaths between 2005 and 2019, placing immense pressure on its emergency care systems (4). Multiple injuries, or polytrauma, are defined as traumatic injuries affecting two or more body systems or organs, with at least one injury being life-threatening (5). These cases are characterized by severe hemorrhage, a high incidence of shock, and a complex pathophysiological response that can rapidly lead to multiple organ dysfunction syndrome (MODS), a major driver of mortality in trauma patients (6).

Modern trauma care follows internationally recognized protocols, such as those in Advanced Trauma Life Support (ATLS), which mandates a structured primary survey using the C-ABCDE approach to identify and manage life-threatening conditions immediately and prioritizes the control of catastrophic hemorrhage (7). Initial stabilization is guided by damage control resuscitation (DCR), a strategy focused on preventing the lethal triad of coagulopathy, acidosis, and hypothermia through permissive hypotension and balanced transfusion protocols (8). Within this framework, skilled nursing is essential for rapid initial assessment, prioritizing interventions, and ensuring timely preparation for subsequent surgical procedures (9). Specifically, the application of validated triage systems by nurses, such as the Emergency Severity Index (ESI), has been shown to more accurately stratify patients by risk, significantly reducing the likelihood of ‘undertriage’ for critically injured patients and ensuring timely resource allocation, thereby indirectly lowering in-hospital mortality (10). In cases of traumatic cardiac arrest, nursing interventions are guided by adjusted advanced cardiac life support (ACLS) principles, where nurses play a core role in assisting with procedures such as bilateral needle decompression for tension pneumothorax and preparing for resuscitative thoracotomy (11). Furthermore, broader, nurse-driven, system-level interventions, including the implementation of rapid response systems, adherence to standardized checklists, and ensuring adequate nurse staffing, are critical components that create a safety network to reduce complications and mortality in critically ill patient populations, including those with trauma (12). However, traditional emergency models are often reactive and respond to physiological deterioration as it occurs. This conventional approach can be fraught with process-related delays in triage, diagnostics, and specialist consultation, which are associated with adverse patient outcomes (13). The sheer complexity of polytrauma can overwhelm a system designed for sequential, problem–response actions, creating a critical need for a more proactive and predictive care model.

Feedforward control theory offers a paradigm shift from reactive to proactive intervention. In a clinical context, it is a predictive control method that involves anticipating potential risks or pathological changes on the basis of initial patient data and intervening preemptively to prevent their occurrence (14). Unlike traditional feedback control, which corrects deviations after they occur, feedforward control focuses on a “prevention first” strategy. This approach has demonstrated significant success in prehospital settings, where a feedforward-based intervention to identify at-risk trauma patients and preemptively manage them for hypothermia was shown to improve care standards and patient stability (15). The core principles of this model—anticipating and acting in advance of a problem—are also fundamental in modern nursing for critically ill patients. For example, in managing traumatic brain injury (TBI), nursing care is centered on proactive neuromonitoring and interventions designed to anticipate and prevent secondary injuries such as cerebral hypoxia and ischemia (16). This anticipatory care model empowers medical teams to move beyond passive monitoring toward active, predictive intervention, which is particularly critical for improving outcomes in the most complex trauma cases (17).

The application of proactive, advanced nursing models in emergency and critical care has been linked to improved clinical outcomes, including a reduced length of stay and a shorter time to consultation, which are vital metrics in the management of polytrauma (13). By applying feedforward control theory to key links in the emergency process, it may be possible to increase treatment speed and increase the success rate of patient rescue. However, there remains a gap in the literature regarding the structured application and analysis of this nursing model, specifically for emergency patients with multiple injuries. Therefore, this study aims to explore the application and effects of key link nursing, which is based on feedforward control theory, in the rescue of this critically ill patient population.

## 2 Data and methods

### 2.1 General data

This study was conducted in the Emergency Department of Suzhou Ninth People’s Hospital, which is a tertiary Grade A hospital in Suzhou, China. The hospital serves an urban and suburban population of approximately 12 million people in the Suzhou metropolitan area and is equipped with a Level I trauma center capable of providing comprehensive care for multiple trauma patients, including advanced life support, multidisciplinary teamwork, and 24/7 emergency surgical services. The Emergency Department has over 60 staff members, including 25 emergency physicians, 30 registered nurses, and 5 specialists in trauma care, and manages more than 55,000 emergency visits annually, with trauma cases accounting for approximately 30% of the total. The facility is supported by modern diagnostic and therapeutic technologies, such as CT, MRI, and ultrasound, and adheres to national guidelines for trauma management. This setting ensures a representative environment for studying emergency multiple trauma care in a high-volume, resource-equipped hospital.

A total of 120 patients with multiple injuries admitted to the emergency department of Suzhou Ninth People’s Hospital from August 2022 to February 2025 were included in this retrospective cohort study. The clinical data of the patients were retrieved from the electronic medical records system and retrospectively analyzed. A consecutive sampling (or convenience sampling) method was employed, including all eligible multiple trauma patients who presented during the study period, to minimize selection bias. The inclusion criteria for patients were as follows: ① aged 20–80 years; ② diagnostic criteria for multiple injuries referred to the relevant standards of “surgery” (18); ③ time from injury to hospital emergency less than 24 h; ④ multiple injuries caused by the same mechanical factor; ⑤ two or more anatomical parts or organs were seriously injured at the same time, and at least one of them was fatal; and ⑤ complete clinical data. The exclusion criteria were as follows: ① patients with minor injuries and no need for treatment; ② patients with multiple injuries caused by acute cerebrovascular disease; ③ patients with interruption of emergency treatment for nonmedical reasons; ④ patients with severe liver or kidney functional damage; ⑤ patients with cardiopulmonary dysfunction and severe mental disorders; and ⑥ patients with combined traumatic cerebral hemorrhage. A total of 120 patients with multiple injuries admitted to the emergency department were divided into groups according to different emergency modes: 46 patients with multiple injuries were included in the conventional group, and 74 patients were included in the feedforward control group. This study was approved by the hospital ethics committee (ethics approval number: 2022-06-C008).

### 2.2 Methods

The design of the feedforward control nursing protocol was predicated on the established principle in trauma care that reduced time to critical interventions is associated with improved survival, particularly in the setting of hemorrhagic shock and severe traumatic brain injury. Our time targets for key processes (e.g., venous access, examination, and transfer to the operating room) were guided by the concepts of the “golden hour” and “platinum 10 min” (19). These concepts emphasize the critical importance of the first hour after injury for definitive care and the first 10 min for initial assessment and life-saving interventions, respectively. Epidemiological and trauma registry studies have consistently shown that a shorter time to operative hemorrhage control and minimum emergency department stay are independent predictors of reduced mortality in polytrauma patients (20). Therefore, the overarching goal of our nursing intervention was to systematically eliminate delays and streamline the entire emergency care pathway to achieve these time-critical milestones as rapidly as possible.

The conventional group received traditional emergency nursing intervention: ① vital signs were monitored continuously, including blood pressure, heart rate, respiratory rate and blood oxygen saturation, and abnormalities were handled in a timely manner. ② Respiratory management: This ensures that the respiratory tract is unobstructed and that oxygen or endotracheal intubation is given when the patient enters the emergency room. ③ Initial injury assessment: emergency medical staff quickly assess the degree of injury and determine its severity through physical examination and medical history collection. ④ Establishment of intravenous access: Rapid establishment of intravenous access to ensure drug and fluid infusion and improve rescue efficiency. ⑤ Wound treatment: Trauma patients undergo thorough debridement to prevent infection and suture or bandage according to the wound condition. Auxiliary examination: X-ray, CT, ultrasound, etc., are used to identify the site of injury; routine blood, biochemical and other examinations are performed; and blood type identification or blood preparation is performed. ⑦ Drug and fluid supplementation: Fluid resuscitation, hemostatics and fluid infusion are given according to the patient’s condition to stabilize vital signs. ⑧ Specialist consultation: Joint consultation with relevant specialists to develop a detailed treatment plan. ⑨ Surgery and outcome: After surgery, the patient will be admitted to the ICU or general ward for postoperative monitoring and rehabilitation care.

The feedforward control group received key-link nursing interventions on the basis of feedforward control theory: (1) Establishment of a professional nursing team: Team formation. A professional nursing team was established, including a multidisciplinary joint nursing team including the head nurse of the emergency department, senior nurses of the emergency department, and relevant specialists. The senior nurses involved in direct patient care for this study group all possessed a minimum of 5 years of clinical experience in emergency or intensive care settings. Team members were responsible for the full control and management of the key links in the rescue process of emergency patients with multiple injuries. The responsibilities were clarified. The head nurse was responsible for the supervision and coordination of overall nursing work and the formulation of nursing plans and work processes; senior nurses were responsible for the implementation and guidance of specific nursing operations and closely observed and evaluated the patient’s condition; and specialists provided professional medical advice and technical support to ensure the scientific validity and effectiveness of the rescue measures. (2) Feedforward control risk assessment: ① Patient condition assessment. Before the patient is admitted to the hospital, the nursing team should immediately conduct a comprehensive and systematic assessment of the patient’s injuries, including the site of injury, severity of injury, vital signs, state of consciousness, potential complications, etc. For example, for patients with craniocerebral injury, the team should focus on assessing their level of consciousness, pupil changes, and signs of increased intracranial pressure; for patients with chest injury, the team should focus on the degree of dyspnea, blood oxygen saturation, and the possibility of pneumothorax or hemothorax. ② Environment and equipment assessment. The environment of the emergency room should be evaluated to ensure that the room is spacious, well lit, and well ventilated to facilitate the smooth progress of rescue operations. Moreover, comprehensive inspection and maintenance of rescue equipment, including electrocardiogram monitors, defibrillators, ventilators, and suction devices, should be conducted to ensure that the equipment is in good condition and ready for use. Various rescue drugs and consumables, such as hemostatic drugs, pressor drugs, and blood transfusion and infusion equipment, should be prepared in advance. ③ Identify potential risk factors. According to the specific situation of the patient, possible risk factors, such as shock, bleeding, infection, and multiple organ dysfunction, can be identified. The probabilities of occurrence and impact of these risk factors are analyzed, and corresponding preventive measures and emergency plans are formulated in advance. (3) Training and assessment of nursing staff: ① Carry out targeted training. Provide systematic training for nursing staff, including emergency first aid knowledge for multiple injuries, key nursing techniques, application of feedforward control theory, risk assessment and prevention measures, etc. This comprehensive training included a specialized component of trauma care, grounded in principles equivalent to those in standardized trauma nursing courses (e.g., Advanced Trauma Care for Nurses—ATCN), to ensure a systematic approach to the polytrauma patient. The professional quality and emergency response capabilities of nursing staff should be improved through theoretical lectures, operational demonstrations, simulation exercises and other forms. ② Strengthen skill training. Focus on training to strengthen nursing staff’s nursing skills in key links, such as wound dressing, hemostasis, fixation, transportation, airway management, and venipuncture. Ensure that nursing staff can master these skills and can operate accurately and quickly in emergency situations. ③ Strict assessment system. Nursing staff are strictly assessed, including their theoretical knowledge and practical skills. Only nursing staff who successfully passed this comprehensive assessment, demonstrating proficiency in both knowledge and trauma-specific skills, were permitted to participate in the rescue of multiple-injury patients in the emergency department for this study to ensure the quality of nursing. (4) Nursing measures in key links: ① Rapid triage and initial treatment. After the patient arrives at the emergency department, the nursing staff performs immediate rapid triage, completing the initial assessment and priority assignment within a target time of ≤5 min, on the basis of the severity of their injuries and in accordance with standard trauma triage protocols (e.g., following ATLS principles). For patients with multiple severe injuries that are life-threatening, the green channel is immediately activated, and rescue treatment is given priority. Moreover, initial treatment, such as maintaining airway patency, stopping bleeding, bandaging wounds, and fixing fractures, is quickly carried out to stabilize the patient’s vital signs. ② Trauma assessment and monitoring. During the rescue process, the nursing staff closely observes the patient’s trauma and promptly detects potential changes in the injury. The patient’s consciousness, pupils, vital signs, wound bleeding, etc., should be regularly assessed and recorded to provide a basis for doctors to adjust the treatment plan. Patients with worsening consciousness disorders, unstable vital signs, or continuous wound bleeding are reported to the doctor in a timely manner and assist in treatment. ③ Emergency nursing operations. The nursing operation procedures used to perform various emergency nursing operations, such as intravenous infusion, blood transfusion, medication, oxygen inhalation, and endotracheal intubation, should be strictly followed. During the operation, attention should be given to aseptic procedures to prevent the occurrence of infection. Moreover, according to the specific situation of the patient, the infusion speed and drug dosage should be reasonably adjusted to ensure the treatment effect. ④ Psychological care and humanistic care. During the rescue process, nursing staff should pay attention not only to patients’ physiological needs but also to patients’ psychological state. For patients with clear consciousness, timely comfort and encouragement should be given to relieve their tension and fear; for family members, they should patiently answer their questions, provide necessary psychological support, help them stay calm, and actively cooperate with the rescue work. (5) Quality control and continuous improvement: ① Establish a quality control system. A complete quality control system is established to monitor the key links in the rescue process of emergency multiple-injury patients. Detailed nursing quality standards and assessment indicators should be formulated, nursing work should be regularly inspected and evaluated, and existing problems and deficiencies should be promptly identified. ② Continuous improvement measures. According to the quality control results, an in-depth analysis of the problems existing in nursing work should be conducted, the causes should be determined, and corresponding improvement measures should be formulated. For example, to address the problem of inadequate preparation of rescue equipment, we strengthened the daily maintenance and management of the equipment and regularly inspected and updated the equipment; to address the problem of unskilled operation of nursing staff, we strengthened training and assessment to improve the professional level of nursing staff. Through continuous quality improvement, we continuously improved the quality of nursing care for emergency patients with multiple injuries and reduced the incidence and mortality of complications.

### 2.3 Evaluation metrics

#### 2.3.1 Treatment time

After treatment, the treatment times, including the time to establish intravenous access, the time to complete the whole-body examination, the time to stay in the emergency department, and the time from the emergency department to the operating room, were compared between the feedforward control group and the conventional group.

#### 2.3.2 Treatment effect

The incidence of MODS and DIC, the referral rate, and the rescue success rate were compared between the feedforward control group and the conventional group.

#### 2.3.3 Injury severity

The injury severity score (ISS) was used to assess the severity of injuries in the feedforward control group and the conventional group. A score of <16 indicated mild injury, 16–25 indicated severe injury, and >25 indicated serious injury.

#### 2.3.4 Patient family satisfaction with nursing

A homemade nursing satisfaction questionnaire was distributed to the families of patients in the feedforward control group and the conventional group to investigate family satisfaction with emergency nursing, including process handling (0–25 points), communication and explanation (0–25 points), rescue skills (0–25 points), and advance preparation (0–25 points). The score ranged from 0 to 100 points. The higher the score is, the greater the degree of satisfaction.

#### 2.3.5 Complications

Compare the incidence of complications such as nerve damage and multiple organ dysfunction in the feedforward control group and the conventional group.

#### 2.3.6 Quality of life

The SF-36 was used to evaluate the quality of life of the feedforward control group and the conventional group. This research team selected five items closely related to the patient’s quality of life, including emotion, physical, social, role and cognitive function, for evaluation. Each dimension is assigned a score of 0--100, and the score is positively correlated with the patient’s quality of life. The evaluation time was 6 months after treatment.

### 2.4 Statistical methods

The research data were analyzed via SPSS 23.0. The measurement data, such as intravenous access time, time to complete systemic examination, and time to stay in the emergency department, were recorded as (x̄ ± *s*). When the data were normally distributed, the t test was used for pairwise comparisons. The counting data, such as the incidence of MODS and DIC, were expressed as [*n* (%)] and tested with the *x*^2^ test. *p* < 0.05 was considered statistically significant.

## 3 Results

### 3.1 Baseline data

**Figure 1:**
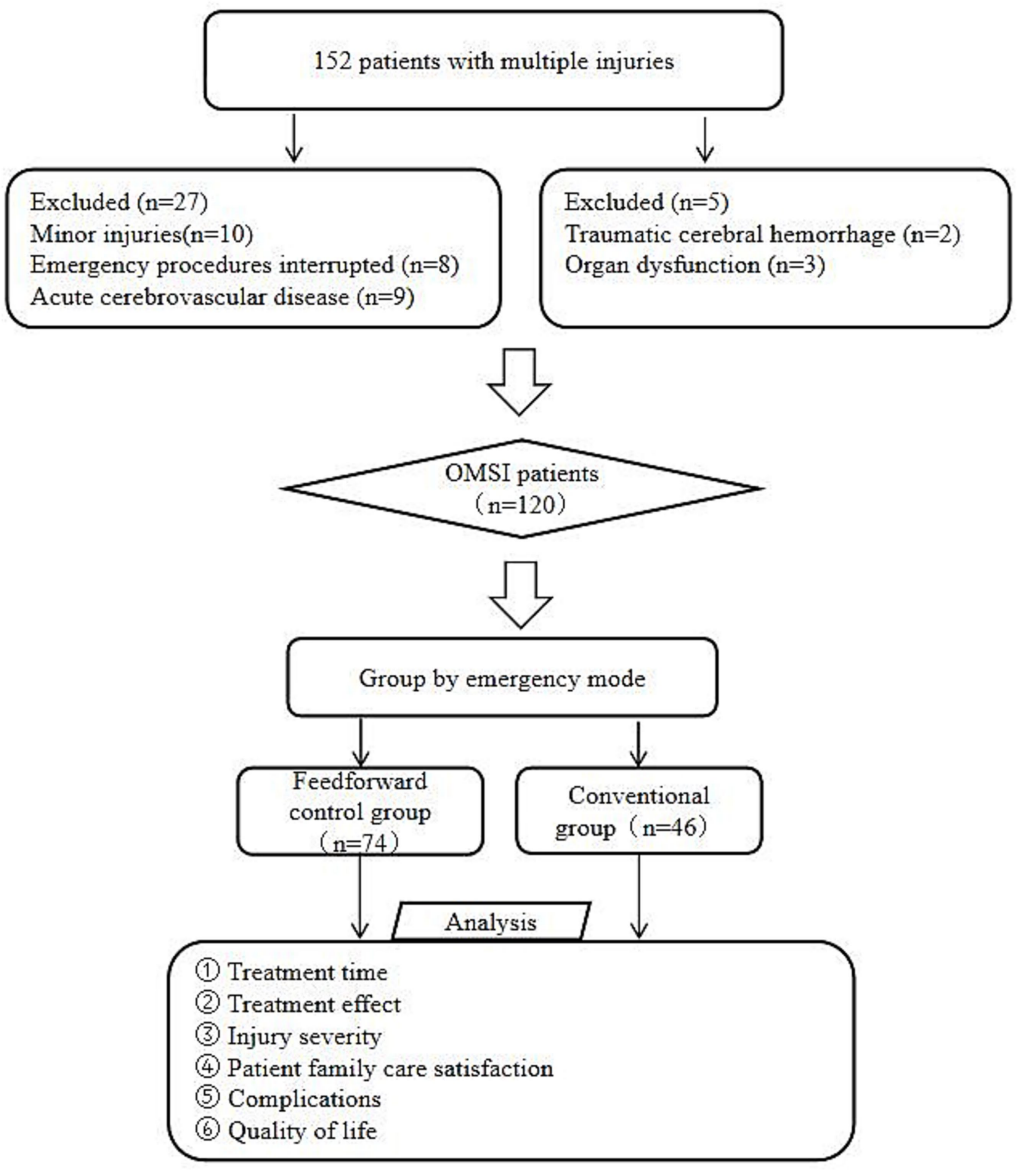
Screening flow chart.

The flow of participants throughout the study is summarized in Figure 1. Baseline characteristics were comparable between the two groups, as shown in Table 1. The baseline data of the feedforward control group, such as age, sex, time from injury to hospital admission, cause of injury, and site of injury, were tested via statistical software and were not significantly different from those of the conventional group (*p* > 0.05).

**Table 1:** Analysis of the baseline data of the two groups.

Material	Feedforward control group (*n* = 74)	Conventional group (*n* = 46)	*x^2^/t*	*p*
Age (years)	42.12 ± 5.13	42.65 ± 5.07	0.452	0.652
Gender (male/female)	38/36	24/22	0.008	0.930
Time to hospitalization after injury (h)	3.54 ± 0.95	3.76 ± 0.92	1.021	0.310
Traumatic causes			1.530	0.465
Falling from height	11	6		
Car accident injury	45	24		
Impact injury	18	16		
Injured area			2.676	0.444
Head and face	21	16		
Limb fractures	23	18		
Chest and abdomen fractures	26	10		
Other	4	2		

### 3.2 Treatment time

The relevant data concerning the treatment time of the two groups are shown in Table 2. The time to establish intravenous access, complete systemic examination, stay time in the emergency department, and time from the emergency room to the operating room in the feedforward control group were significantly shorter than those in the conventional group (*p* < 0.05).

**Table 2:** Comparison of treatment times between the two groups of patients (x̄ ± s).

Index	Feedforward control group (*n* = 74)	Conventional group (*n* = 46)	*t*	*p*
Time to establish intravenous access (min)	11.98 ± 2.14	15.76 ± 3.02	6.549	<0.001
Time to complete full body examination (min)	24.11 ± 3.54	37.44 ± 4.56	14.655	<0.001
Emergency room stay time (min)	61.77 ± 14.76	71.22 ± 16.15	2.6	0.009
Time from emergency department to operating room (min)	70.43 ± 19.88	81.10 ± 20.22	2.323	0.023

### 3.3 Treatment effect

The data related to the treatment effects of the two groups are shown in Table 3. The incidence rates of MODS and DIC and the referral rate in the feedforward control group were significantly lower than those in the conventional group, and the success rate of rescue was significantly greater than that in the conventional group (*p* < 0.05).

**Table 3:** Analysis of treatment effects in the two groups [*n* (%)].

Grouping	Number of cases	MODS	DIC	Referral	Successful rescue
Feedforward control group	74	6 (8.11)	3 (4.05)	0 (0.00)	69 (93.24)
Conventional group	46	12 (26.09)	9 (19.57)	4 (8.70)	36 (78.26)
*x^2^*		7.192	7.583	6.657	5.822
*p*		0.007	0.006	0.010	0.016

### 3.4 Trauma severity

The data related to the severity of trauma in the two groups are shown in Table 4. The severity of trauma in the feedforward control group and the conventional group significantly improved after treatment. Specifically, after treatment, the proportion of severe injuries and serious injuries in the feedforward control group and the conventional group decreased, and the proportion of minor injuries increased. Compared with before rescue, the difference was significant (*p* < 0.05). In addition, the proportion of minor injuries in the feedforward control group increased significantly after treatment compared with that in the conventional group, and the proportion of severe injuries decreased significantly compared with that in the conventional group (*p* < 0.05).

**Table 4:** Comparison of trauma severity between the two groups [*n* (%)].

Grouping	Minor injuries		Seriously injured		Serious injury	
	Before rescue	After treatment	Before rescue	After treatment	Before rescue	After treatment
Feedforward control group (*n* = 74)	9 (12.16)	62 (83.78)^*^	39 (57.70)	8 (10.81)^*^	26 (35.14)	4 (5.41)^*^
Conventional group (*n* = 46)	4 (8.70)	28 (60.87)^*^	24 (51.61)	13 (29.03)^*^	18 (39.13)	5 (10.87)^*^
*x^2^*	0.353	7.944	0.003	5.983	0.195	1.221
*p*	0.552	0.005	0.955	0.014	0.659	0.269

vs. before rescue, *p < 0.05.

### 3.5 Patient family satisfaction with care

The evaluation of nursing care by the family members of the two groups of patients is shown in Table 5. The process handling, communication explanation, rescue skills, prepreparation scores and overall nursing satisfaction scores of the feedforward control group were significantly higher than those of the conventional group (*p* < 0.05).

**Table 5:** Comparison of the nursing satisfaction of family members in the two groups of patients (x̄ ± s).

Grouping	Process handling	Communication explanation	Rescue skills	Advance preparation	Satisfaction with care
Feedforward control group (*n* = 74)	22.34 ± 1.56	22.51 ± 1.78	23.13 ± 1.22	22.66 ± 1.50	90.64 ± 6.06
Conventional group (*n* = 46)	19.43 ± 1.46	18.03 ± 1.44	20.33 ± 1.40	19.88 ± 1.11	77.67 ± 5.41
*t*	8.330	11.778	9.442	8.886	9.713
*p*	<0.001	<0.001	<0.001	<0.001	<0.001

### 3.6 Complications

According to the data in Table 6, in the feedforward control group, there were 3 cases of nerve injury, 2 cases of large area necrosis, and 3 cases of shock, for a total of 8 cases, and the total incidence of complications was 10.81%. In the conventional group, there were 3 cases of nerve injury, 3 cases of large area necrosis, 5 cases of shock, and 1 case of multiple organ dysfunction, for a total of 12 cases, and the total incidence of complications was 26.09%. The difference between the two groups was obvious (*p* < 0.05).

**Table 6:** Comparison of complication rates between the two groups [n (%)].

Grouping	Nerve damage	Extensive tissue necrosis	Shock	Multiple organ dysfunction	Overall incidence
Feedforward control group (*n* = 74)	3 (4.05)	2 (2.70)	3 (4.05)	0 (0.00)	8 (10.81)
Conventional group (*n* = 46)	3 (6.52)	3 (6.52)	5 (10.87)	1 (2.17)	12 (26.09)
*x^2^*					4.766
*p*					0.029

### 3.7 Quality of life

The SF-36 scores of the two groups of patients at 6 months after treatment are shown in Table 7. The scores of the emotional, physical, social, role and cognitive function dimensions in the feedforward control group were significantly greater than those in the conventional group (p < 0.05).

**Table 7:** Comparison of SF-36 scores between the two groups (x̄ ± s).

Grouping	Emotional function	Physical function	Social function	Role function	Cognitive function
Feedforward control group (*n* = 74)	85.44 ± 5.33	82.12 ± 6.90	79.89 ± 4.55	81.90 ± 6.72	80.19 ± 6.71
Conventional group (*n* = 46)	72.13 ± 4.12	69.33 ± 5.67	71.78 ± 4.12	74.90 ± 5.51	72.48 ± 6.64
*t*	11.836	8.634	8.051	4.855	5.027
*p*	<0.001	<0.001	<0.001	<0.001	<0.001

## 4 Discussion

Multiple injuries have become one of the main causes of death in emergency patients due to complex injury mechanisms, severe injuries, rapid disease changes, and many infections and complications. A core driver of this mortality is the development of systemic complications such as trauma-induced coagulopathy and multiple organ dysfunction syndrome (21). For emergency multiple-injury patients, life is life-long. Early implementation of rescue and key nursing interventions can significantly reduce mortality and ensure the safety of patients. During the treatment process, providing patients with effective nursing interventions is particularly important to improve the success rate of rescue (22). This aligns with a global trend toward implementing more structured, proactive, and systems-based nursing approaches to enhance trauma care quality (23). While the traditional emergency nursing model can meet patients’ needs to a degree, it often loses time, failing to meet the demands of severe multiple injury cases and negatively impacting outcomes (24). Therefore, the emergency care process for patients with multiple injuries needs to be optimized and improved. The feedforward control model functions as this optimization by requiring nurses to foresee risk factors and implement corresponding measures preemptively. The advantage is that it shifts the nursing paradigm from reactive to proactive, a necessary evolution given the complexity of polytrauma. It aims to shorten rescue times, proactively prepare for treatment, improve communication, and ultimately enhance patient survival and quality of life, which is consistent with the goals of modern emergency care models that emphasize resilience and foresight among nursing staff (25).

In this study, the time to establish intravenous access, complete systemic examination, stay in the emergency department, and time from the emergency department to the operating room in the feedforward control group were significantly shorter (*p* < 0.05), indicating that key link nursing based on feedforward control theory can shorten the patient’s treatment time. This is because the feedforward approach front loads the trauma response by using predefined protocols and clarifying team roles, which is critical for efficiency (26). In this model, after group discussion, relevant improvement processes were formulated to make the patient’s emergency process more convenient, the green channel smoother, the rescue process more orderly and efficient, and multiple departments working together to provide emergency protection. By anticipating needs such as prenotifying radiology and surgical teams and using standardized communication, the model proactively addresses potential bottlenecks before they occur. This principle is mirrored in successful quality improvement strategies for trauma systems globally, which consistently lead to faster patient care (27). This finding is further supported by studies demonstrating that optimized emergency nursing procedures (28) and the use of cognitive aids such as the ABCDE checklist (29) significantly increase the efficiency and reliability of the resuscitation process.

Second, this study revealed that the incidence rates of MODS and DIC, the referral rate, the proportion of severe injuries and the complication rate in the feedforward control group were significantly lower than those in the conventional group. This is a critical finding, as it suggests that the model’s proactive nature directly mitigates severe pathophysiological consequences. This finding aligns with evidence showing that structured, multidisciplinary trauma care models significantly reduce such complications (30). Nursing care based on feedforward control theory focuses on preemptive actions such as early preparation of rescue items and advanced notification to various departments. This functions as a practical application of the principles of damage control resuscitation, which proactively manages the ‘lethal triad’ of coagulopathy, acidosis, and hypothermia to prevent downstream consequences (31). The focus on early and effective hemorrhage control, a cornerstone of European trauma guidelines, is central to preventing subsequent complications such as MODS and DIC (32). Effective nursing plays a standardized and critical role in hemorrhage management and infection control within these protocols (33), further validating the success of our intervention, which leads to better patient outcomes similar to those observed in other enhanced trauma management programs (34).

In addition, this study revealed that the process handling, communication and explanation, rescue skills, prepreparation score, total nursing satisfaction score, and SF-36 scores of the feedforward control group were significantly greater than those of the conventional group (*p* < 0.05). This highlights the vital importance of structured, proactive communication within the often chaotic environment of trauma resuscitation. Applying feedforward control principles to family interaction involves anticipating their informational and emotional needs. This proactive communication is supported by robust evidence; for example, structured communication tools in the ICU have been proven to significantly increase family satisfaction (35). This finding shows that the key link nursing, which is based on feedforward control theory, can also achieve the maximum utilization of medical care resources and provide high-quality clinical nursing services. The nurse’s role as the primary communicator is consistently identified as the single most significant factor influencing trauma patient satisfaction (36). Our findings are bolstered by studies on nurse-led family support interventions, which demonstrate that proactive updates and emotional support empower families and improve their satisfaction with the care process (37, 38). This aligns with broader healthcare trends that advocate for cocreating models of care with community and family involvement to improve service delivery and satisfaction (39). Ultimately, this contributes to a better nurse patient relationship and strengthens the overall healthcare system (40).

This study has several limitations that should be considered. First, the sample size of 120 patients may limit the statistical power and generalizability of the findings, preventing them from fully reflecting the value of feedforward control-based nursing in a wider population. This sample size over the approximately 2.5-year study period can be attributed to our stringent inclusion criteria, which were designed to create a homogeneous cohort of true polytrauma patients (ISS ≥ 16, at least one life-threatening injury), and to the fact that the Suzhou metropolitan area is served by multiple tertiary hospitals with advanced trauma capabilities, leading to a natural distribution of trauma patients. Second, the single-center, retrospective design from only one hospital means that the results may be influenced by local factors such as regional characteristics, specific medical protocols, and patient demographics, which limits the extrapolation of the findings. Future studies should aim to overcome these limitations by conducting prospective, multicenter, large-scale clinical trials to provide more robust evidence for saving the lives of polytrauma patients and improving their prognosis.

## 5 Conclusion

Key link nursing, which is based on feedforward control theory, has significant advantages in the emergency care of multiple trauma patients. This approach substantially shortens critical rescue timeframes, including intravenous access establishment and completion of full-body examination. It markedly reduces the incidence of serious complications such as MODS and DIC while significantly improving the rescue success rate. Furthermore, the intervention led to better trauma severity outcomes posttreatment, with more patients achieving minor injury status. The feedforward control method also effectively lowers the overall complication rate and enhances both family satisfaction with care and patients’ long-term quality of life across multiple dimensions. These findings support the implementation of this proactive nursing strategy as an effective approach to optimize trauma care processes and improve patient outcomes.

## Data Availability

The raw data supporting the conclusions of this article will be made available by the authors, without undue reservation.
